# Supplementary material for: SARS-CoV-2-specific cellular and humoral immunity after bivalent BA.4/5 COVID-19-vaccination in previously infected and non-infected individuals
Source: Nat Commun. 2024 Apr 9;15:3077. doi: 10.1038/s41467-024-47429-8 (PMC11004149; doi:10.1038/s41467-024-47429-8)
Supplement: Supplementary file 1 — Supplementary information [file 41467_2024_47429_MOESM1_ESM.docx]

# Supplementary information

# SARS-CoV-2-specific cellular and humoral immunity after bivalent BA.4/5 COVID-19-vaccination in previously infected and non-infected individuals

Rebecca Urschel^1^; Saskia Bronder^1^; Verena Klemis^1^; Stefanie Marx^1^; Franziska Hielscher^1^; Amina Abu-Omar^1^; Candida Guckelmus^1^; Sophie Schneitler^2^; Christina Baum^3^; Sören L. Becker^2^; Barbara C. Gärtner^2^; Urban Sester^4^; Leonardo Martinez^5^; Marek Widera^6^; Tina Schmidt^1^; and Martina Sester^1,7*^

^1^Department of Transplant and Infection Immunology, Saarland University; 66421 Homburg, Germany; ^2^Institute of Medical Microbiology and Hygiene, Saarland University; 66421 Homburg, Germany; ^3^Occupational Health Care Center, Saarland University; 66421 Homburg, Germany; ^4^Department of Nephrology, SHG-Klinikum Völklingen, 66333 Völklingen, Germany; ^5^Boston University, School of Public Health, Department of Epidemiology, Boston, MA, USA. ^6^Institute for Medical Virology, University Hospital Frankfurt, Goethe University Frankfurt, Germany; ^7^Center for Gender-specific Biology and Medicine (CGBM), Saarland University; 66421 Homburg, Germany.

# Supplementary tables and figures

## Table S1: Clinical characteristics of breakthrough infections in individuals with and without infection prior to bivalent vaccination.

|  | **All** | **Infected** | **Non-infected** | p-value |
| --- | --- | --- | --- | --- |
|  | **n=126** | **n=64** | **n=62*** |  |
| **Follow-up observation time** [days after vaccination] median (IQR) | 146 (10.3) | 146 (8.0) | 146 (10.3) | 0.210^‡^ |
| **Breakthrough infections** n, (%) |  |  |  | 0.045^†^ |
| yes | 25 (19.84%) | 9 (14.06%) | 16 (25.81%) |  |
| no | 97 (76.98%) | 51 (79.69%) | 46 (74.19%) |  |
| unknown | 4 (3.17%) | 4 (6.25%) | 0 (0.00%) |  |
| **Breakthrough infections** | **n=25** | **n=9** | **n=16** |  |
| **Time to infection** [days after vaccination] median (IQR) | 129 (64.5) | 137 (45.5) | 128 (87.8) | 0.192^‡^ |
| **Incidence rate** [cases/100.000 person-days (95% CI)] | 149 (97-221) | 106 (49-202) | 194 (111-315) | 0.145 |
| **Vaccine regimen** |  |  |  | >0.999 |
| mRNA | 12 (48.00%) | 4 (44.44%) | 8 (50.00%) |  |
| Vector/ mRNA combination | 13 (52.00%) | 5 (55.56%) | 8 (50.00%) |  |
| **Symptom score**^$^ n (IQR) |  |  |  | 0.500^†^ |
| asymptomatic | 1 (4.00%) | 0 (0.00%) | 1 (6.25%) |  |
| mild | 14 (56.00%) | 5 (55.56%) | 9 (56.25%) |  |
| moderate | 8 (32.00%) | 4 (44.44%) | 4 (25.00%) |  |
| moderate-severe | 2 (8.00%) | 0 (0.00%) | 2 (12.50%) |  |
| hospitalized | 0 (0.00%) | 0 (0.00%) | 0 (0.00%) |  |
| **Duration of symptoms**^$^ n (%) |  |  |  | 0.170^†^ |
| 0 days | 1 (4.00%) | 0 (0.00%) | 1 (6.25%) |  |
| 1-2 days | 8 (32.00%) | 4 (44.44%) | 4 (25.00%) |  |
| 3-6 days | 7 (28.00%) | 4 (44.44%) | 3 (18.75%) |  |
| 7 days or more | 9 (36.00%) | 1 (11.11%) | 8 (50.00%) |  |

^*^follow-up information on one individual in the non-infected group was not available; p-values refer to comparisons between individuals with and without prior infection. ^‡^Mann-Whitney test; ^#^Fisher´s test; ^†^X^2^ test (all two-sided); ^$^Scoring was based on self-assessment using a questionnaire. Source data are provided as a source data file.

## Table S2: Multivariate logistic regression analysis for all individuals, previously non-infected and infected individuals.

| All individuals | Parental | | | BA.4/5 | | | BA.1 | | | BA.2 | | |
| --- | --- | --- | --- | --- | --- | --- | --- | --- | --- | --- | --- | --- |
|  | OR | CI | p-value | OR | CI | p-value | OR | CI | p-value | OR | CI | p-value |
| Neutralizing Ab | **0.70** | **0.49-0.98** | **0.039** | **0.65** | **0.48-0.85** | **0.003** | **0.75** | **0.57-0.96** | **0.025** | **0.65** | **0.47-0.86** | **0.004** |
| CD4 T cells | 0.33 | 0.05-2.12 | 0.235 | 0.32 | 0.05-2.15 | 0.243 | 0.17 | 0.02-1.27 | 0.094 | 0.43 | 0.07-2.46 | 0.341 |
| age | 1.00 | 0.96-1.03 | 0.785 | 0.99 | 0.96-1.03 | 0.731 | 1.00 | 0.96-1.03 | 0.892 | 0.99 | 0.96-1.03 | 0.647 |
| sex | 0.8103 | 0.28-2.18 | 0.685 | 0.74 | 0.24-2.09 | 0.579 | 0.77 | 0.26-2.10 | 0.614 | 0.89 | 0.29-2.50 | 0.822 |
| previously non-infected individuals | Parental | | | BA.4/5 | | | BA.1 | | | BA.2 | | |
|  | OR | CI | p-value | OR | CI | p-value | OR | CI | p-value | OR | CI | p-value |
| Neutralizing Ab | 0.61 | 0.33-1.01 | 0.070 | **0.49** | **0.28-0.77** | **0.005** | **0.65** | **0.42-0.95** | **0.03** | **0.54** | **0.32-0.82** | **0.009** |
| CD4 T cells | **0.03** | **0.001-0.75** | **0.041** | **0.04** | **0.001-0.69** | **0.037** | **0.03** | **0.001-0.63** | **0.04** | 0.21 | 0.01-2.47 | 0.243 |
| age | 0.95 | 0.89-1.00 | 0.071 | 0.94 | 0.88-1.00 | 0.080 | 0.96 | 0.90-1.01 | 0.14 | 0.95 | 0.89-1.01 | 0.097 |
| sex | 2.31 | 0.52-10.42 | 0.266 | 1.73 | 0.33-8.73 | 0.504 | 2.20 | 0.46-10.32 | 0.31 | 2.53 | 0.51-12.72 | 0.246 |
| previously infected individuals | Parental | | | BA.4/5 | | | BA.1 | | | BA.2 | | |
|  | OR | 95% CI | p-value | OR | 95% CI | p-value | OR | 95% CI | p-value | OR | 95% CI | p-value |
| Neutralizing Ab | 1.10 | 0.56-2.36 | 0.783 | 0.86 | 0.47-1.47 | 0.466 | 0.9698 | 0.61-1.56 | 0.896 | 0.75 | 0.39-1.37 | 0.356 |
| CD4 T cells | 1.34 | 0.11-22.25 | 0.822 | 3.31 | 0.15-91.33 | 0.458 | 1.129 | 0.05-26.7 | 0.940 | 2.42 | 0.11-60.67 | 0.576 |
| age | 1.05 | 0.99-1.11 | 0.133 | 1.05 | 0.99-1.11 | 0.117 | 1.043 | 0.99-1.11 | 0.147 | 1.04 | 0.99-1.11 | 0.150 |
| sex | 0.28 | 0.04-1.39 | 0.154 | 0.27 | 0.03-1.40 | 0.157 | 0.274 | 0.04-1.36 | 0.148 | 0.27 | 0.03-1.41 | 0.159 |

Multivariate logistic regression analysis was used to assess the relationship between immunological parameters (spike-specific CD4 T cells and neutralizing antibodies) and occurrence of breakthrough infections. adjusted for age and gender in all individuals (n=127), previously non-infected (n=63), and previously infected individuals (n=64). T cell parameters were log10 transformed, neutralizing antibodies were log2 transformed before analysis; variance inflation factors (VIF) were below 1.4. Shown are odds ratios (OR). 95% confidence intervals (95% CI). and p-values. with significant values marked in bold. Two-sided tests were applied. Source data underlying these analyses are provided as a source data file.

## Table S3: Receiver operating characteristics analysis for models with neutralizing antibodies and CD4 T cell responses.

|  | | | All individuals (n=127) | | | Previously non-infected individuals (n=63) | | | Previously infected individuals (n=64) | | |
| --- | --- | --- | --- | --- | --- | --- | --- | --- | --- | --- | --- |
| Model | Parameter | Strain type | AUC | 95% CI | p-value | AUC | 95% CI | p-value | AUC | 95% CI | p-value |
| Base model | Neutralizing Ab | parental | 0.66 | 0.54-0.78 | 0.008 | 0.71 | 0.56-0.85 | 0.013 | 0.50 | 0.31-0.70 | 0.969 |
| Add to base modell | CD4 T cells | parental | 0.67 | 0.55-0.80 | 0.008 | 0.77 | 0.63-0.91 | 0.001 | 0.53 | 0.36-0.71 | 0.768 |
| Base model | Neutralizing Ab | BA.4/5 | 0.69 | 0.57-0.82 | 0.003 | 0.77 | 0.63-0.90 | 0.002 | 0.50 | 0.31-0.69 | >0.999 |
| Add to base modell | CD4 T cells | BA.4/5 | 0.71 | 0.58-0.83 | 0.002 | 0.81 | 0.69-0.92 | 0.0002 | 0.60 | 0.43-0.77 | 0.346 |
| Base model | Neutralizing Ab | BA.1 | 0.64 | 0.51-0.78 | 0.028 | 0.70 | 0.55-0.86 | 0.016 | 0.52 | 0.31-0.73 | 0.844 |
| Add to base modell | CD4 T cells | BA.1 | 0.67 | 0.54-0.80 | 0.009 | 0.74 | 0.60-0.88 | 0.004 | 0.52 | 0.30-0.73 | 0.875 |
| Base model | Neutralizing Ab | BA.2 | 0.70 | 0.58-0.82 | 0.002 | 0.73 | 0.60-0.87 | 0.006 | 0.55 | 0.34-0.77 | 0.610 |
| Add to base modell | CD4 T cells | BA.2 | 0.71 | 0.60-0.84 | 0.001 | 0.78 | 0.66-0.90 | 0.0009 | 0.57 | 0.37-0.77 | 0.510 |

Uni- and multivariate logistic regression analysis was used to test whether neutralizing antibodies predict breakthrough infection and whether adding T cells within this model improves prediction of breakthrough infection; T cell parameters were log10 transformed, neutralizing antibodies were log2 transformed before analysis; variance inflation factors (VIF) were below 1.4. Shown are areas under the curve (AUC), 95% confidence intervals (95% CI), and p-values. Two-sided tests were applied. Source data underlying these analyses are provided as a source data file.

## Table S4: Multivariate logistic regression interaction analysis of prior infection and predictors for breakthrough infections.

| **All individuals** | **Breakthrough infection** | | |
| --- | --- | --- | --- |
|  | OR | CI | p-value |
| IgG | 0.39 | 0.01-10.14 | 0.565 |
| IgG * prior infection | 0.30 | 0.006-14.57 | 0.544 |
| Nab parental | 1.03 | 0.54-2.14 | 0.924 |
| Nab parental * prior infection | 0.55 | 0.23-1.21 | 0.155 |
| Nab BA.1 | 0.95 | 0.59-1.52 | 0.814 |
| Nab BA.1 * prior infection | 0.67 | 0.37-1.21 | 0.197 |
| Nab BA.2 | 0.76 | 0.40-1.35 | 0.360 |
| Nab BA.2 * prior infection | 0.73 | 0.35-1.55 | 0.407 |
| Nab BA.4/5 | 0.87 | 0.48-1.49 | 0.638 |
| Nab BA.4/5 * prior infection | 0.58 | 0.28-1.21 | 0.145 |
| CD4 parental | 1.28 | 0.11-19.22 | 0.849 |
| **CD4 parental * prior infection** | **0.02** | **0.0003-0.69** | **0.039** |
| CD4 BA.1 | 0.90 | 0.04-17.71 | 0.947 |
| CD4 BA.1 * prior infection | 0.03 | 0.0003-1.61 | 0.089 |
| CD4 BA.2 | 1.53 | 0.09-32.22 | 0.773 |
| CD4 BA.2 * prior infection | 0.06 | 0.001-2.02 | 0.127 |
| CD4 BA.4/5 | 2.52 | 0.17-49.5 | 0.643 |
| **CD4 BA.4/5 * prior infection** | **0.0099** | **0.0002-0.44** | **0.022** |
| CD8 parental | 1.28 | 0.42-4.15 | 0.664 |
| CD8 parental * prior infection | 0.68 | 0.16-2.73 | 0.585 |
| CD8 BA.1 | 1.92 | 0.58-6.96 | 0.295 |
| CD8 BA.1 * prior infection | 0.52 | 0.11-2.21 | 0.379 |
| CD8 BA.2 | 1.85 | 0.53-7.04 | 0.342 |
| CD8 BA.2 * prior infection | 0.61 | 0.13-2.74 | 0.519 |
| CD8 BA.4/5 | 1.54 | 0.44-5.81 | 0.504 |
| CD8 BA.4/5 * prior infection | 0.69 | 0.15-2.99 | 0.619 |
| age | 1.03 | 0.98-1.09 | 0.249 |
| age * prior infection | 0.95 | 0.88-1.01 | 0.125 |
| sex | 0.37 | 0.05-1.69 | 0.239 |
| sex * prior infection | 4.03 | 0.54-39.18 | 0.189 |

Multivariate logistic regression analysis was used to assess the relationship between parameters such as IgG, neutralizing antibodies, spike-specific CD4 and CD8 T cells, age and sex, and occurrence of breakthrough infections with prior infection added as an interaction term (prior infection [yes] as a reference); shown are odds rations (OR), 95% confidence intervals (95% CI), and p-values with significant values marked in bold. Two-sided tests were applied. Source data underlying these analyses are provided as a source data file.

## Table S5: Antibodies used for flow-cytometric analyses.

| **Antigen** | **Conjugate** | **Clone** | **Isotype** | **Reactivity** | **Dilution** | **Catalogue number** |
| --- | --- | --- | --- | --- | --- | --- |
| CD4 | APC-H7 | SK3 | IgG1 k | mouse anti-human | 1:33.3 | 641398 |
| CD8 | PerCP | SK1 | IgG1 k | mouse anti-human | 1:12.5 | 345774 |
| CD69 | PE-Cy7 | L78 | IgG1 k | mouse anti-human | 1:33.3 | 335792 |
| CTLA-4 | APC | BNI3 | IgG2a k | mouse anti-human | 1:50 | 555855 |
| IFNγ | FITC | 4S.B3 | IgG1 k | mouse anti-human | 1:100 | 554551 |
| IL-2 | PE | MQ1-17H12 | IgG2a k | rat anti-human | 1:12.5 | 559334 |
| TNF | V450 | MAb11 | IgG1 k | mouse anti-human | 1:20 | 561311 |

All antibodies from BD, Heidelberg, Germany.

## Figure S1


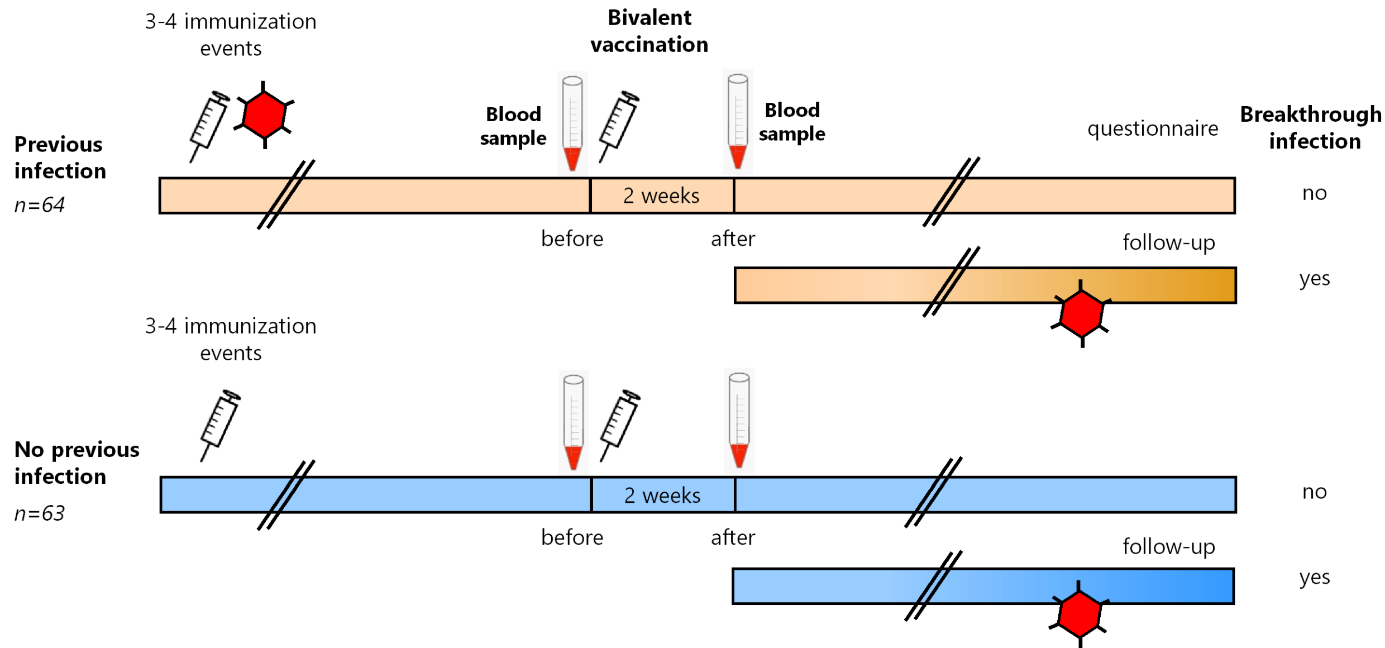


**Figure S1: Schematic representation of study design.** Individuals after 3-4 immunization events with (orange symbols, n=64) and without prior infection (blue symbols, n=63) were recruited. Blood sampling was performed before and after bivalent vaccination. All individuals were followed up for occurrence of breakthrough infection using a questionnaire.

## Figure S2


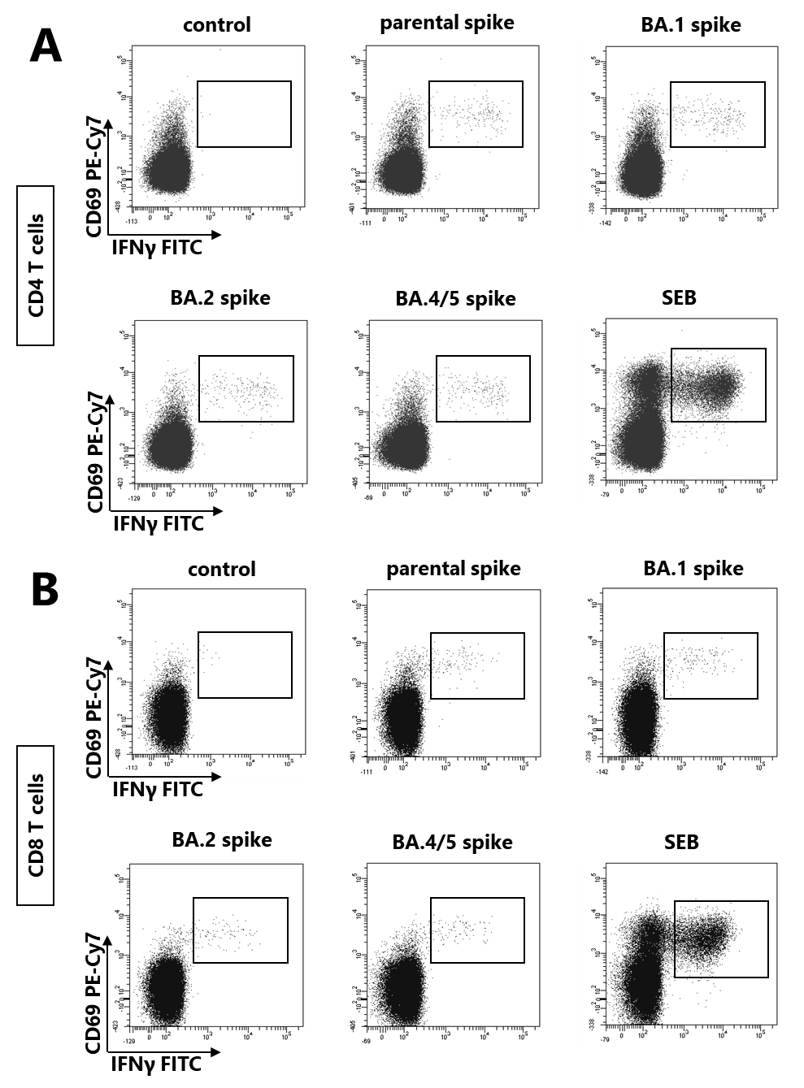


**Figure S2: Representative analysis of spike- and SEB-reactive CD4 and CD8 T cells.** Stimulations of whole blood from a previously infected individual were carried out with negative control (DMSO diluent) as well as with overlapping peptide pools derived from spike of the parental SARS-CoV-2 and Omicron subvariants BA.1, BA.2, BA. 4/5 or with SEB as a polyclonal stimulus. **(A)** CD4 and **(B)** CD8 T cells co-expressing the activation marker CD69 and the cytokine IFNγ are shown. Boxes were used as gates to quantify the percentage of CD69^+^/IFNγ^+^ CD4 or CD8 T cells; SEB, *Staphylococcus aureus* Enterotoxin B.

## Figure S3


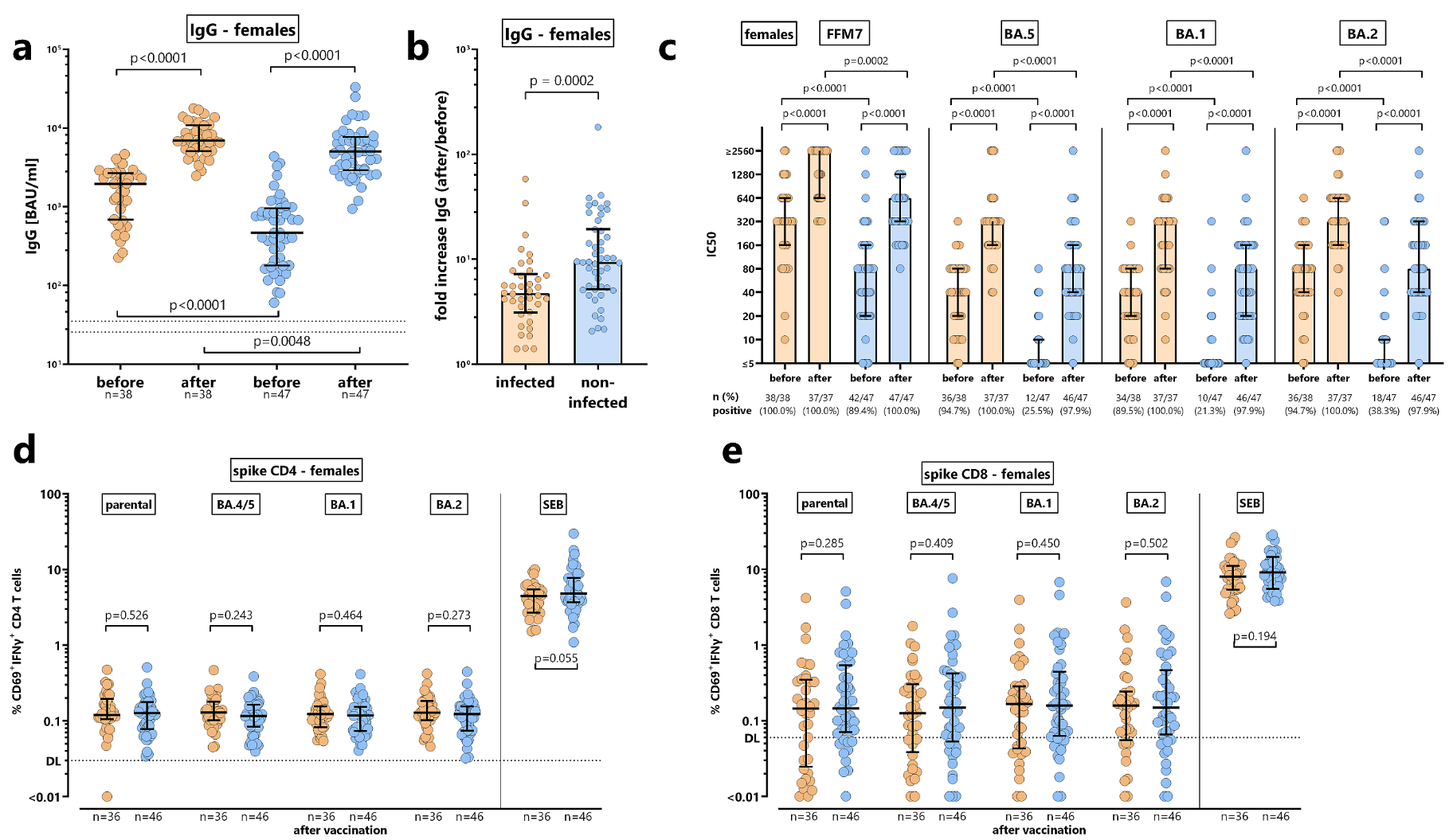


**Figure S3: Immunogenicity in female study participants. (a)** Spike-specific IgG levels (in BAU/ml) towards the parental SARS-CoV-2 spike protein were determined from individuals with (orange symbols, n=38) and without prior infection (blue symbols, n=47) before and after bivalent vaccination. Statistical analysis (two-sided) was performed using the paired t-test (before/after) or the non-parametric Mann-Whitney test for between-group comparisons at baseline and after vaccination. **(b)** The fold increase in spike-specific IgG levels was determined for individuals with (n=38) and without prior infection (n=47) and compared using Mann-Whitney test (two-sided). **(c)** Neutralizing activity of antibodies towards authentic parental SARS-CoV-2 (FFM7) and Omicron subvariants were determined in infected and non-infected individuals using a microneutralization assay, and differences were calculated using the paired t-test (before/after) or the non-parametric Mann-Whitney test (both two-sided) for between-group comparisons at baseline and after vaccination. Numbers of tested individuals per group are indicate. **(d)** CD4 and **(e)** CD8 T cells towards parental spike and towards the spike-protein of the Omicron subvariants BA.4/5, BA.1 and BA.2 were determined after bivalent vaccination (infected n=36 (n=63 for BA.2), non-infected n=46). SEB-reactive CD4 and CD8 T cell levels were quantified as positive controls. Statistical analysis was performed using the Mann-Whitney test (two-sided). Stippled lines denote detection limits (0.03% for CD4 T cells and 0.06% for CD8 T cells). Lines indicate medians and interquartile ranges. SEB, Staphylococcus aureus Enterotoxin B. Numbers refer to biologically independent samples examined in one experiment per individual per time point. Source data are provided as a source data file.

## Figure S4

**
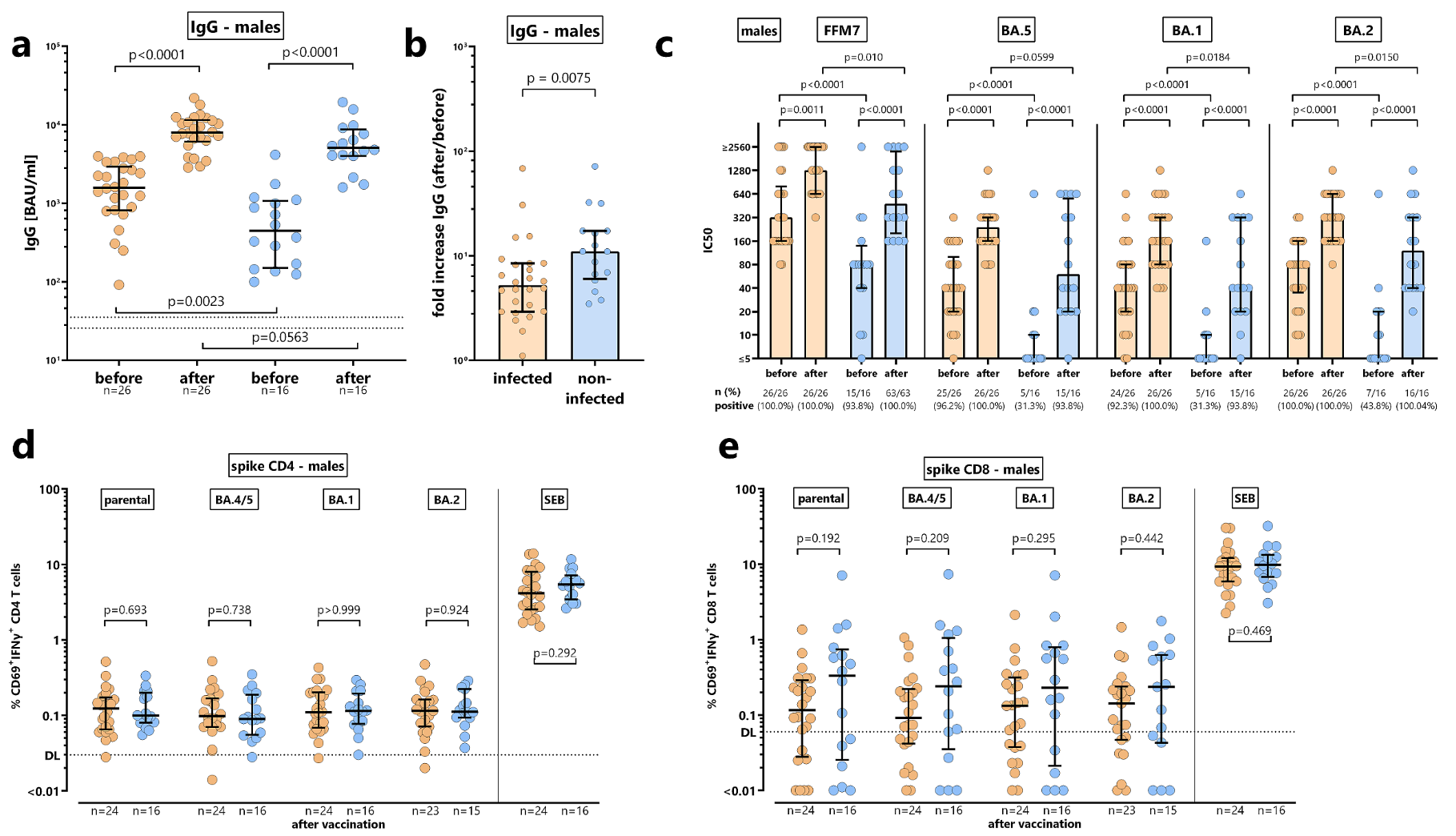
**

**Figure S4: Immunogenicity in male study participants. (a)** Spike-specific IgG levels (in BAU/ml) towards the parental SARS-CoV-2 spike protein were determined from individuals with (orange symbols, n=26) and without prior infection (blue symbols, n=16) before and after bivalent vaccination. Statistical analysis (two-sided) was performed using the paired t-test (before/after) or the non-parametric Mann-Whitney test for between-group comparisons at baseline and after vaccination. **(b)** The fold increase in spike-specific IgG levels was determined for individuals with (n=26) and without prior infection (n=16) and compared using Mann-Whitney test (two-sided). **(c)** Neutralizing activity of antibodies towards authentic parental SARS-CoV-2 (FFM7) and Omicron subvariants were determined in infected and non-infected individuals using a microneutralization assay, and differences were calculated using the paired t-test (before/after) or the non-parametric Mann-Whitney test (both two-sided) for between-group comparisons at baseline and after vaccination. Numbers of tested individuals per group are indicate. **(d)** CD4 and **(e)** CD8 T cells towards parental spike and towards the spike-protein of the Omicron subvariants BA.4/5, BA.1 and BA.2 were determined after bivalent vaccination (infected n=24 (n=23 for BA.2), non-infected n=16 (15 for BA.2). SEB-reactive CD4 and CD8 T cell levels were quantified as positive controls. Statistical analysis was performed using the Mann-Whitney test (two-sided). Stippled lines denote detection limits (0.03% for CD4 T cells and 0.06% for CD8 T cells). Lines indicate medians and interquartile ranges. SEB, Staphylococcus aureus Enterotoxin B. Numbers refer to biologically independent samples examined in one experiment per individual per time point. Source data are provided as a source data file.

## Figure S5


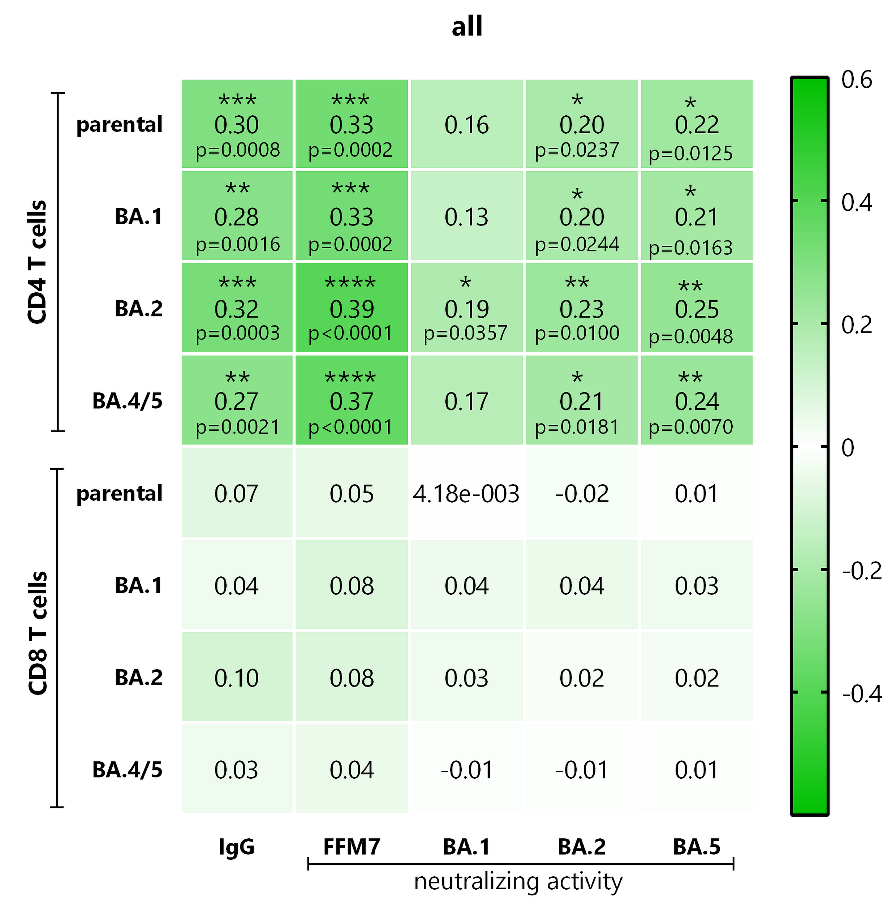


**Figure S5: Correlation between vaccine-induced cellular and humoral immunity.** Correlation matrix between vaccine-induced spike-specific CD4 or CD8 T cells with IgG levels and neutralizing activities towards parental SARS-CoV2 and Omicron subvariants in all individuals (n=125 biologically independent samples examined in one experiment per individual). Correlation coefficients were calculated according to two-tailed Spearman and displayed using a color code, and p-values (including stars denoting levels of statistical significance) are indicated. Separate analyses of data for individuals with and without prior infection is shown in figure 4 of the main manuscript. Source data underlying theses analyses are provided as a source data file.

## Figure S6

**Figure S6: CTLA-4 expression of parental spike-specific and SEB-reactive CD4 and CD8 T cells before and after bivalent vaccination in previously infected and non-infected individuals. (a)** Specific CD4 and **(b)** CD8 T cells towards the parental spike and SEB-reactive T cells were analyzed for expression of CTLA-4, which is expressed as median fluorescence intensity (MFI). All samples (from all individuals) were analyzed, but to ensure robust statistics, data were restricted to samples with at least 20 CD69+ IFNγ+ CD4 or CD8 T cells (with sample size indicated in the figures). Lines represent medians with interquartile ranges. Differences between the groups were calculated using the Mann Whitney test. Numbers refer to biologically independent samples examined in one experiment per individual. Source data are provided as a source data file.

## Figure S7


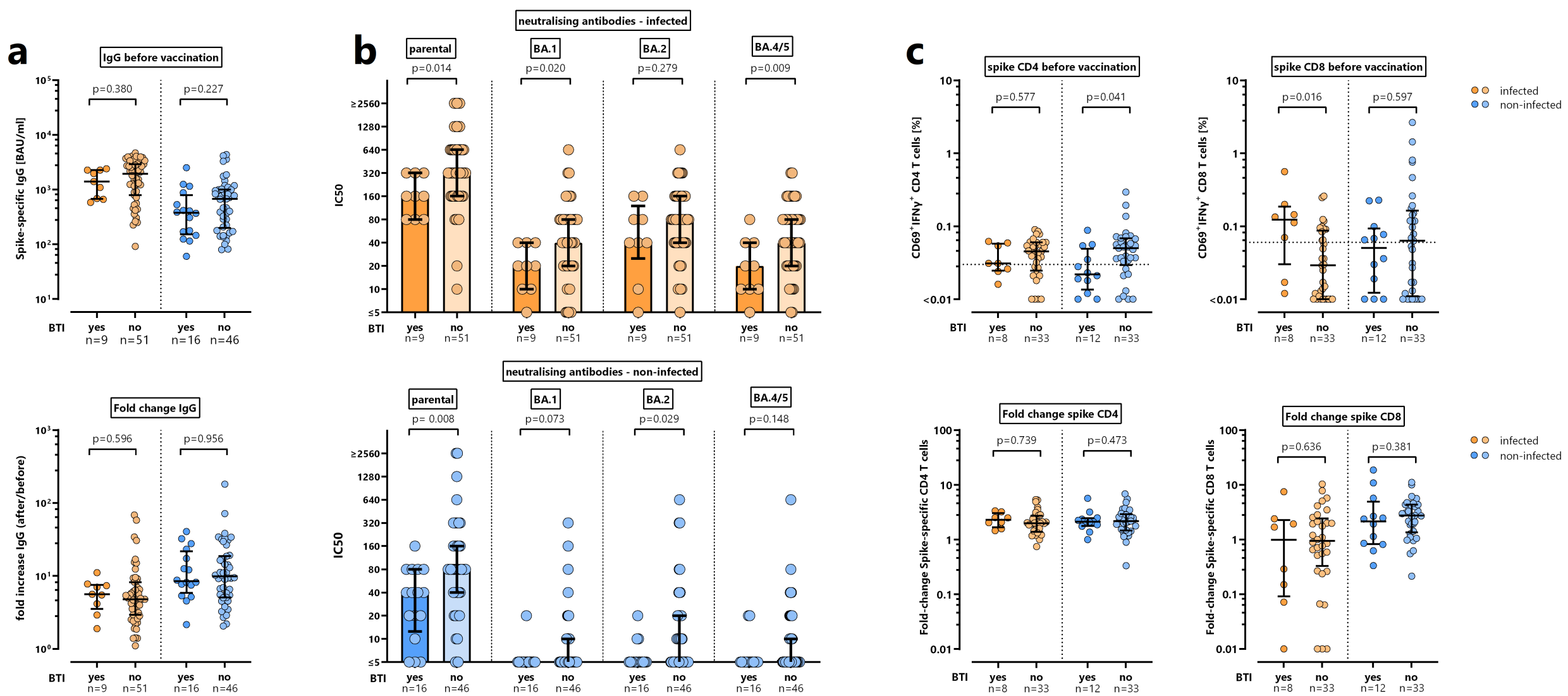


**Figure S7: Pre-vaccination SARS-CoV-2 spike-specific IgG, neutralizing antibodies, and spike-specific T cell responses in individuals with and without prior infection subdivided according to the occurrence of breakthrough infections. (a)** SARS-CoV-2 spike-specific IgG in Ab-binding units (BAU) /ml were quantified by ELISA from plasma samples of non-infected (n=62) and infected (n=60) individuals before bivalent vaccination, and groups were stratified based on occurrence of subsequent breakthrough infection. Moreover, the fold-change from pre to post vaccination was determined (lower panels). **(b)** Neutralizing antibody titers towards the parental SARS-CoV-2 (FFM), and Omicron variants of concern BA.1, BA.2 and BA.5 were determined using authentic virus. Neutralisation was tested from plasma samples in reciprocal dilutions resulting in neutralisation titres that represent 50% virus neutralisation (IC50) of the respective SARS-CoV-2 variants. Bar plots are shown for comparison of Ab-mediated neutralisation before bivalent vaccination in non-infected (blue) individuals (n=62) and infected (orange) individuals (n=60). **(c)** T cell responses against SARS-CoV-2 spike protein in non-infected (n=45 and infected (n=41) individuals were analyzed. Spike-specific CD4 and CD8 T cells were identified by flow cytometry based on co-expression of CD69 and IFNγ after antigen-specific stimulation of whole blood, normalized by subtraction of reactivity towards the negative control DMSO. Dotted lines indicate cut-offs for spike-specific CD4 T cells (0.03%) and CD8 T cells (0.06%). Moreover, the fold-change from pre to post vaccination was determined (lower panels). Statistical analyses were carried out using the Mann-Whitney test. Lines or bars indicate medians and interquartile ranges. Numbers refer to biologically independent samples examined in one experiment per individual. Source data are provided as a source data file.

## Figure S8


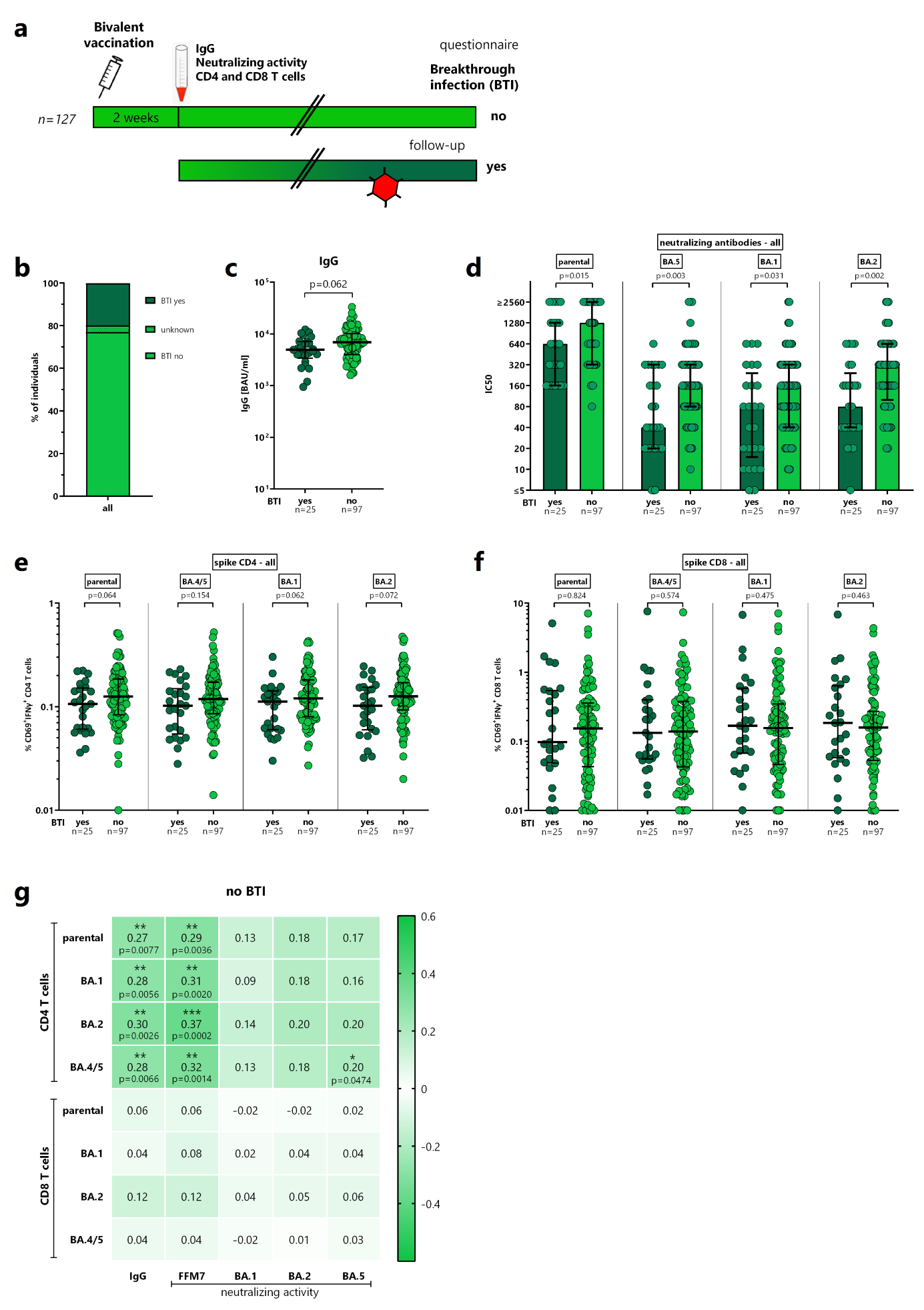


**Figure S8: Vaccine-induced humoral and cellular immunity in individuals with and without breakthrough infections. (a)** Schematic outline of the study design. All study participants were followed up until March 2023 for development of breakthrough infections based on self-reporting using a questionnaire. Immune parameters in the following panels refer to results two weeks after the bivalent vaccination in all individuals (n=126). **(b)** Percentage of breakthrough infections (BTI) (n=126). Bivalent vaccine-induced spike-specific **(c)** IgG levels, **(d)** neutralizing antibody activity, **(e)** CD4 T cells, and **(f)** CD8 T cells stratified for individuals with and without subsequent breakthrough infection. Statistical analysis was performed using the Mann-Whitney test. **(g)** Matrix of correlations between vaccine-induced spike-specific CD4 or CD8 T cells with IgG levels and neutralizing activities towards parental SARS-CoV2 and Omicron subvariants in all individuals without breakthrough infections. BTI, breakthrough infection. Separate analyses of data in panels b-f for individuals with and without prior infection is shown in figure 6 of the main manuscript. Lines or bars in panels b-f indicate medians and interquartile ranges. Numbers refer to biologically independent samples examined in one experiment per individual. Source data are provided as a source data file.

## Figure S9


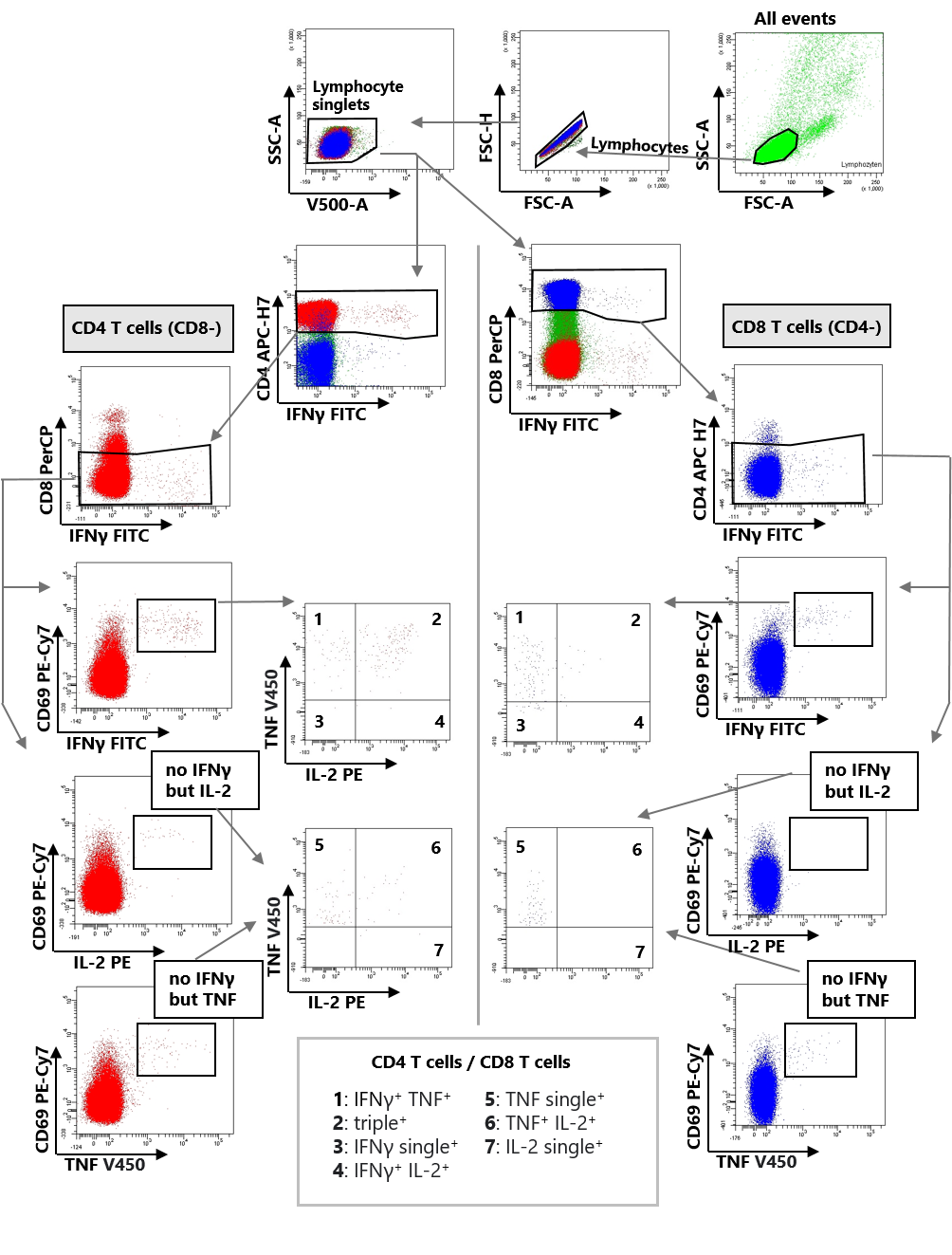


**Figure S9: Gating strategy for identification of antigen-specific CD4 and CD8 T cells after stimulation.** Lymphocytes were identified among total events by backgating of CD4 and/or CD8 positive cells combined with signals for size (FSC) and granularity (SSC). Hight and area signals of FSC were used to exclude doublets. The gating strategy to identify CD4 T cells (left side) or CD8 T cells (right side) co-expressing the activation marker CD69 and the cytokines IFNγ, IL-2 or TNF are shown. Boxes were used as gates to quantify the percentage of CD69^+^/IFNγ^+^ CD4 or CD8 T cells as shown in figures 3, 6e/f, S7c, S8e/f. The median fluorescence intensity (MFI) of CTLA-4 was determined from CD4 T cells (left side or CD8 T cells (right side) co-expressing the activation marker CD69 and the cytokines IFNγ. Data are shown in figures 5a and S6. Moreover, Boolean gating for cytokine profiling is shown that was used to identify subpopulations of CD4 or CD8 T cells expressing all three cytokines (triple^+^), two cytokines or one cytokine only (shown in figure 5b).
