# Supplementary material for: SARS-CoV-2-specific cellular and humoral immunity after bivalent BA.4/5 COVID-19-vaccination in previously infected and non-infected individuals
Source: Nat Commun. 2024 Apr 9;15:3077. doi: 10.1038/s41467-024-47429-8 (PMC11004149; doi:10.1038/s41467-024-47429-8)
Supplement: Supplementary file 3 — Reporting Summary [file 41467_2024_47429_MOESM3_ESM.pdf]

nature portfolio

Corresponding author(s): Martina Sester

Last updated by author(s): 13.3.2024

Reporting Summary

Nature Portfolio wishes to improve the reproducibility of the work that we publish. This form provides structure for consistency and transparency in reporting. For further information on Nature Portfolio policies, see our Editorial Policies and the Editorial Policy Checklist.

Statistics

For all statistical analyses, confirm that the following items are present in the figure legend, table legend, main text, or Methods section.

n/a	Confirmed	
☐	☒	The exact sample size (*n*) for each experimental group/condition, given as a discrete number and unit of measurement
☐	☒	A statement on whether measurements were taken from distinct samples or whether the same sample was measured repeatedly
☐	☒	The statistical test(s) used AND whether they are one- or two-sided<br>*Only common tests should be described solely by name; describe more complex techniques in the Methods section.*
☐	☒	A description of all covariates tested
☐	☒	A description of any assumptions or corrections, such as tests of normality and adjustment for multiple comparisons
☐	☒	A full description of the statistical parameters including central tendency (e.g. means) or other basic estimates (e.g. regression coefficient) AND variation (e.g. standard deviation) or associated estimates of uncertainty (e.g. confidence intervals)
☐	☒	For null hypothesis testing, the test statistic (e.g. *F*, *t*, *r*) with confidence intervals, effect sizes, degrees of freedom and *P* value noted<br>*Give P values as exact values whenever suitable.*
☒	☐	For Bayesian analysis, information on the choice of priors and Markov chain Monte Carlo settings
☒	☐	For hierarchical and complex designs, identification of the appropriate level for tests and full reporting of outcomes
☒	☐	Estimates of effect sizes (e.g. Cohen's *d*, Pearson's *r*), indicating how they were calculated

Our web collection on statistics for biologists contains articles on many of the points above.

Software and code

Policy information about availability of computer code

Data collection	BD FACSdiva software 6.1.3
Data analysis	BD FACSdiva software 6.1.3<br>GraphPad Prism software 10.0.3

For manuscripts utilizing custom algorithms or software that are central to the research but not yet described in published literature, software must be made available to editors and reviewers. We strongly encourage code deposition in a community repository (e.g. GitHub). See the Nature Portfolio guidelines for submitting code & software for further information.

Data

Policy information about availability of data

All manuscripts must include a data availability statement. This statement should provide the following information, where applicable:

- Accession codes, unique identifiers, or web links for publicly available datasets
- A description of any restrictions on data availability
- For clinical datasets or third party data, please ensure that the statement adheres to our policy

Table 1, Figures 1-6, Tables S1-S4, and Figures S3-S8 have associated raw data. Source data are provided with this paper, and data are available in a public repository (https://zenodo.org/records/10815331). As age may be subject to confidentiality, data in the repository refer to age groups.

Research involving human participants, their data, or biological material

Policy information about studies with human participants or human data. See also policy information about sex, gender (identity/presentation), and sexual orientation and race, ethnicity and racism.

Reporting on sex and gender	Information on sex was collected based on self-reporting, and both sexes were considered without any restrictions. Overall numbers are as follows: 64 previously infected immunocompetent individuals and 63 previously non-infected immunocompetent individuals were included in the study. Among the infected individuals 26 were males and 38 were females. Among the non-infected individuals 16 were males and 47 were females. Further characteristics of the study population are shown in table 1 and table S1 of the manuscript.
Reporting on race, ethnicity, or other socially relevant groupings	All individuals were whites.
Population characteristics	127 immunocompetent individuals were included in the study. Individuals were enrolled prior to receiving vaccination with the bivalent vaccine Comirnaty Original/Omicron BA.4-5, BioNTech/Pfizer as per German recommendations. Among the individuals, 64 had a history of prior infection (46.5±13.7 years of age, 26 males, 38 females) and 63 did not have a history of infection (52.6±12.7 years of age, 16 males, 47 females). Further characteristics of the study population is shown in table 1 and table S1 of the manuscript.
Recruitment	Prospective recruitment; all individuals were enrolled prior to vaccination as convenience sampling, assignment of the vaccine was determined as per current guidelines and availability of the bivalent vaccine (only Comirnaty Original/Omicron BA.4-5, BioNTech/Pfizer was available). We do not see any evidence for a potential self-selection bias or other biases that may be present.
Ethics oversight	Ethikkommission der Ärztekammer des Saarlandes

Note that full information on the approval of the study protocol must also be provided in the manuscript.

Field-specific reporting

Please select the one below that is the best fit for your research. If you are not sure, read the appropriate sections before making your selection.

☒ Life sciences ☐ Behavioural & social sciences ☐ Ecological, evolutionary & environmental sciences

For a reference copy of the document with all sections, see nature.com/documents/nr-reporting-summary-flat.pdf

Life sciences study design

All studies must disclose on these points even when the disclosure is negative.

Sample size	observational study with sample size according to availability, real-world recruitment of vaccinated individual at Saarland University Medical campus and a public vaccination campaign (convenience sampling).
Data exclusions	none
Replication	Experimental procedures were pre-established before. Sufficient cells and plasma volumes were available to be analysed on each sample. Within each sample, no replicates were performed due to limited sample material.
Randomization	not applicable; individuals were recruited in an observational study with convenience samples being collected.
Blinding	During sample processing and analysis of primary data, the investigators were blinded to individual characteristics of study subjects (i.e. prior infection, no infection, sex, age, etc.)

Reporting for specific materials, systems and methods

We require information from authors about some types of materials, experimental systems and methods used in many studies. Here, indicate whether each material, system or method listed is relevant to your study. If you are not sure if a list item applies to your research, read the appropriate section before selecting a response.

Materials & experimental systems

n/a	Involved in the study
☐	☒ Antibodies
☐	☒ Eukaryotic cell lines
☒	☐ Palaeontology and archaeology
☒	☐ Animals and other organisms
☒	☐ Clinical data
☒	☐ Dual use research of concern
☒	☐ Plants

Methods

n/a	Involved in the study
☒	☐ ChIP-seq
☐	☒ Flow cytometry
☒	☐ MRI-based neuroimaging

Antibodies

Antibodies used

anti-CD4 mouse anti-human (BD Biosciences, APC-H7, clone SK3, cat. 641398, Dilution 1:100 for unstimulated samples and 1:33.3 for stimulated samples)
anti-CD8 mouse anti-human (BD Biosciences, PerCP, clone SK1, cat. 345774, Dilution 1:12.5)
anti-CD28 mouse anti-human (BD Biosciences, purified, clone L293, cat. 348040, 1µg/ml)
anti-CD49d mouse anti-human (BD Biosciences, purified, clone 9F10, cat. 555501, 1µg/ml)
anti-CD69 mouse anti-human (BD Biosciences, PE-Cy7, clone L78, cat. 335792, Dilution 1:33.3)
anti-IFNγ mouse anti-human (BD Biosciences, FITC, clone 4S.B3, cat. 554551, Dilution 1:100)
anti-IL-2 rat anti-human (BD Biosciences, PE, clone MQ1-17H12, cat. 559334, Dilution 1:12.5)
anti-TNFα mouse anti-human (BD Biosciences, V450, clone Mab11, cat. 561311, Dilution 1:20)
anti-CTLA-4 mouse anti-human (BD Biosciences, APC, clone BNI3, cat. 555855, Dilution 1:50)

Validation

All antibodies were separately titrated in house to evaluate best concentrations for discrimination of negative and positive cells. Finally, panels including defined concentration of antibodies were tested together to confirm that identified antibody concentrations are also sufficient when compensations are required.

SK3: The CD4 antibody, clone SK3, is derived from the hybridization of mouse NS-1 myeloma cells with spleen cells from BALB/c mice immunized with human peripheral blood T lymphocytes. The CD4 antibody recognizes a 55-kilodalton (kDa) glycoprotein that is present on T-helper/inducer lymphocytes and monocytes. Application: Flow cytometry; Source: https://www.bdbiosciences.com/en-de/products/reagents/flow-cytometry-reagents/clinical-discovery-research/single-color-antibodies-ruo-gmp/apc-h7-mouse-anti-human-cd4.641398

SK1: CD8 is intended for in vitro diagnostic use in the identification of cells expressing CD8 antigen. Application: Flow cytometry; Source: https://www.bdbiosciences.com/en-de/products/reagents/flow-cytometry-reagents/clinical-diagnostics/single-color-antibodies-asr-ivd-ce-ivd/cd8-percp.345774

L293: The CD28 antibody, clone L293, is derived from hybridization of Sp2/0-Ag14 mouse myeloma cells with spleen cells from BALB/c mice immunized with the HPB-ALL T-cell line. The CD28 antigen, a disulfide-linked homodimeric glycoprotein, Mr 44 kilodaltons (kd), is a cell-adhesion molecule (CAM) and functions as the ligand for CD80 (B7-1) and CD86 (B7-2) antigens, which are present on activated B lymphocytes, monocytes, and dendritic cells. Interaction of the CD28 antigen with CD80 or CD86 antigens, or both, co-stimulates CD2 and CD3 antigen/T-cell antigen receptor (TCR)–dependent T-cell–mediated proliferation and cytotoxicity. Application: Flow cytometry (RUO GMP); Source: https://www.bdbiosciences.com/en-de/products/reagents/flow-cytometry-reagents/clinical-discovery-research/single-color-antibodies-ruo-gmp/purified-mouse-anti-human-cd28.348040

9F10: The 9F10 monoclonal antibody specifically reacts with the integrin α4 chain, that is expressed as a heterodimer with either of two β integrin subunits, β1 (CD29) or β7. The α4β1 integrin (VLA-4) is expressed on lymphocytes, monocytes, thymocyte
s, NK cells, and several B- and T-cell lines, and mediates binding to VCAM-1 (CD106) and the CS-1 region of fibronectin. The α4β7 integrin has a similar tissue distribution, except it is found on only a small subpopulation of thymocytes. Integrin α4β7 also binds fibronectin and VCAM-1, and has been shown in the mouse to preferentially bind the mucosal vascular addressin molecule, MAdCAM-1. This antibody is useful for studies of the expression by and function of cells that express α4 chain-containing integrins. This clone cross-reacts with a subset of peripheral blood lymphocytes, monocytes, and some granulocytes of baboon and both rhesus and cynomolgus macaque monkeys. The distribution on leukocytes is similar to that observed with human peripheral blood leukocytes. Application: Flow cytometry (Routinely Tested); Source: https://www.bdbiosciences.com/en-de/products/reagents/flow-cytometry-reagents/research-reagents/single-color-antibodies-ruo/purified-na-le-mouse-anti-human-cd49d.555501

L78: The CD69 antibody, clone L78, is derived from hybridization of mouse Sp2/0-Ag14 myeloma cells with lymph node cells from BALB/c mice immunized with a CD8+ alloantigen–directed cytotoxic T-lymphocyte (CTL) cell line. The CD69 antibody recognizes a very early human activation antigen that is a disulfide-bonded homodimer consisting of Mr 60-kilodalton (kDa) polypeptides with one or two N-linked oligosaccharides. Application: Flow cytometry (RUO GMP); Source: https://www.bdbiosciences.com/en-de/products/reagents/flow-cytometry-reagents/clinical-discovery-research/single-color-antibodies-ruo-gmp/pe-cy-7-mouse-anti-human-cd69.335792

4S.B3: The 4S.B3 monoclonal antibody specifically binds to interferon-γ (IFN-γ). The immunogen used to generate this hybridoma was partially purified human IFN-γ obtained from supernatants of human PBMC stimulated with Staphylococcus aureus. Interferon-γ (IFN-γ) is a potent multifunctional cytokine that is produced by several activated cell types including NK, NKT, CD4+TCRαβ+, CD8+TCRαβ+, and TCRγδ+ T cells. IFN-γ exerts its biological effects through specific binding to the high-affinity IFN-γ Receptor Complex comprised of IFN-γRα (CD119) and IFN-γRβ subunits. In addition to its antiviral effects, IFN-γ upregulates a number of lymphoid cell functions

including the antimicrobial and antitumor responses of macrophages, NK cells, and neutrophils. In addition, IFN-γ can exert strong regulatory influences on the proliferation, differentiation, and effector responses of B cell and T cell subsets. These influences can involve IFN-γ's capacity to boost MHC class I and II expression by antigen-presenting cells as well as to direct effects on B cells and T cells themselves. Human IFN-γ is a 14-18 kDa glycoprotein containing 143 amino acid residues. Clone 4S.B3 also cross-reacts with a cytoplasmic component of peripheral blood CD3+ lymphocytes of baboon, and both rhesus and cynomolgus macaque monkeys following five-hour treatment with phorbol myristic acetate (PMA) and Ca++ ionophore (A23187) in the presence of monensin. The staining pattern of 4S.B3 in CD3+ cells is similar to that observed with peripheral blood T lymphocytes from normal human donors. This reagent is useful for intracellular immunofluorescent staining for flow cytometric analysis to identify and enumerate IFN-γ + cells within a mixed cell population. Application: Intracellular staining (flow cytometry, Routinely Tested); Source: https://www.bdbiosciences.com/en-de/products/reagents/flow-cytometry-reagents/research-reagents/single-color-antibodies-ruo/fitc-mouse-anti-human-ifn.554551

MQ1-17H12: The MQ1-17H12 monoclonal antibody specifically binds to the multifunctional cytokine, human Interleukin-2 (IL-2). IL-2 is produced by activated T cells and has multiple functions that can affect the growth, proliferation, differentiation and survival of many different target cell types including T cells, B cells, NK cells, monocytes and macrophages. The immunogen used to generate the MQ1-17H12 hybridoma was purified recombinant human IL-2 protein. The MQ1-17H12 antibody reportedly neutralizes the biological activity of human IL-2. Application: Intracellular staining (flow cytometry, Routinely Tested); Source: https://www.bdbiosciences.com/en-de/products/reagents/flow-cytometry-reagents/research-reagents/single-color-antibodies-ruo/pe-rat-anti-human-il-2.559334

MAb11: The MAb11 monoclonal antibody specifically binds to human tumor necrosis factor (TNF, also known as TNF-α) protein. TNF is an efficient juxtacrine, paracrine and endocrine mediator of inflammatory and immune functions. It regulates the growth and differentiation of a variety of cell types. TNF is cytotoxic for transformed cells when in conjunction with IFN-γ. It is secreted by activated monocytes/macrophages and other cells such as B cells, T cells and fibroblasts. The immunogen used to generate the MAb11 hybridoma was recombinant human TNF. The MAb11 antibody has been reported to crossreact with Rhesus Macaque TNF. Application: Intracellular staining (flow cytometry, Routinely Tested); Source: https://www.bdbiosciences.com/en-de/products/reagents/flow-cytometry-reagents/research-reagents/single-color-antibodies-ruo/v450-mouse-anti-human-tnf.561311

The BNI3 monoclonal antibody specifically binds to the human cytolytic T lymphocyte-associated antigen (CTLA-4), also known as CD152. CTLA-4 is transiently expressed on activated CD28+ T cells and binds to CD80 and CD86 present on antigen presenting cells (APC) with high avidity. This interaction appears to deliver a negative regulatory signal to the T cell. Recent reports indicate that CTLA-4 is also expressed on B cells when cultured with activated T cells, suggesting a role for CTLA-4 in the regulation of B-cell response. Immobilized BNI3 antibody enhances T-cell proliferation induced by antibody-mediated crosslinking of CD3 and CD28. Recent studies have shown that CD152 can be expressed by regulatory T (Treg) cells. After cellular fixation and permeabilization, the BNI3 antibody can stain intracellular CD152 expressed in T cells including Treg cells. Clone BNI3 was studied in the VI Leukocyte Typing Workshop. Source: APC Mouse Anti-Human CD152 (bdbiosciences.com)

Eukaryotic cell lines

Policy information about cell lines and Sex and Gender in Research

Cell line source(s)	Original A549 cells (male) were obtained from DSMZ (#ACC107). The human ACE2-dTomato and TMPRSS2-BFP expression cassettes were stably integrated using the Spleeping Beauty Transposase (Widera et al. 2021; https://doi.org/10.3389/fmicb.2021.701198). Cells were FACS-sorted for high expression of both transgenes.
Authentication	The cell line was authenticated and confirmed as part of routine regulatory checks. The expression of all relevant markers was performed using FACS analysis.
Mycoplasma contamination	We confirm that all cell lines were tested negative for mycoplasma contamination.
Commonly misidentified lines (See ICLAC register)	No misidentified cell lines were used in this study.

Plants

Seed stocks	n.a.
Novel plant genotypes	n.a.
Authentication	n.a.

Flow Cytometry

Plots

Confirm that:

☒ The axis labels state the marker and fluorochrome used (e.g. CD4-FITC).

☒ The axis scales are clearly visible. Include numbers along axes only for bottom left plot of group (a 'group' is an analysis of identical markers).

☒ All plots are contour plots with outliers or pseudocolor plots.

☒ A numerical value for number of cells or percentage (with statistics) is provided.

Methodology

Sample preparation	Whole blood analysis
Instrument	BD FACS Canto II
Software	BD FACSdiva software 6.1.3<br>GraphPad Prism software 10.0.3
Cell population abundance	All blood samples were tested without further processing (i.e. no purification or cell sorting).
Gating strategy	Gating strategies are outlined in dedicated figure (figure S9).<br><br>Gating of antigen-reactive T cells:<br>Lymphocytes were identified among total events by backgating of CD4 and/or CD8 positive cells combined with signals for size (FSC) and granularity (SSC). Hight and area signals of FSC were used to exclude doublets. CD4 T-cells were identified among single cells by CD4 positive and CD8 negative signals. Likewise, CD8 T-cells were defined as T-cells being CD8 positive and CD4 negative. Antigen-reactive cells were identified as CD4 or CD8 T-cells co-expressing the activation marker CD69 and the cytokines IFNγ, IL-2 and/or TNF. Boundaries between “cytokine-negative” and “cytokine-positive” was defined using negative control stimulations.<br>For additional analysis of cytokine expression profiles, NOT Boolean Gating was used to identify all CD4 T-cells that were not CD69+IFNγ+. Among these cells CD69+IL-2+ and CD69+TNF+ CD4 T-cells were gated and combined using OR Boolean Gating. CD69+IL-2+ and/or CD69+TNF+ CD4 T-cells were divided into IL-2 single, TNF single or IL-2+TNF+ cells.

☒ Tick this box to confirm that a figure exemplifying the gating strategy is provided in the Supplementary Information.
